# NURSING STUDENTS’ EXPERIENCES REGARDING PHYSICAL RESTRAINTS: AN INTEGRATIVE REVIEW

**DOI:** 10.1093/geroni/igad104.3499

**Published:** 2023-12-21

**Authors:** Juh Hyun Shin, Eun-Hi Kong

**Affiliations:** George Washington University, Ashburn, Virginia, United States; Gachon University, Incheon, Gyeonggi, Republic of Korea

## Abstract

Many harmful effects related to the use of physical restraints have been reported and restraint-free care has been advocated for in many countries. However, the prevalence of physical restraint use is still high in many care settings around the world. There has been no systematic review study focused on nursing students regarding physical restraints. This study aimed to critically review the existing studies targeting nursing students related to physical restraint using an integrative review method. This study was registered in PROSPERO(CRD42023414410). Eleven electronic databases were searched by two authors: CINAHL, PubMed, EMBASE, PsycINFO, ERIC, Education Source, SCOPUS, RISS, DBpia, Cochrane, and KoreaMed from the earliest year to 2023. A total of 19 studies including diverse research designs were selected. For quality appraisal, both the Mixed Methods Appraisal Tool (MMAT) and the Joanna Briggs Institutes (JBI) checklist were employed. Most studies were conducted in acute clinical care settings. Many studies used a cross-sectional study design, convenience sampling method, small number of participants, and weak measurements. There was a lack of experimental studies, mixed methods studies, and financially funded studies. The main outcomes were nursing students’ attitude, knowledge, perception, or practice regarding physical restraints. This integrative review provided new knowledge and understanding about nursing students’ experiences regarding physical restraints. Future studies must use more robust research design, sample size, and measurements with research funding. This study showed that more research and education targeting nursing students are needed to achieve restraint-free care.

